# *Trypanosoma cruzi*: Gene Expression Surveyed by Proteomic Analysis Reveals Interaction between Different Genotypes in Mixed In Vitro Cultures

**DOI:** 10.1371/journal.pone.0095442

**Published:** 2014-04-18

**Authors:** Alexandre Machin, Jenny Telleria, Jean-Paul Brizard, Edith Demettre, Martial Séveno, Francisco José Ayala, Michel Tibayrenc

**Affiliations:** 1 Unité Mixte de Recherche, Institut de Recherche pour le Développement/Centre National de la Recherche Scientifique/Universités Montpellier 1 and 2, Génétique et Evolution des Maladies Infectieuses, n° 5290, Maladies Infectieuses et Vecteurs Ecologie, Génétique, Evolution et Contrôle (MIVEGEC) Institut de Recherche pour le Développement, Montpellier, France; 2 Unité Mixte de Recherche, Institut de Recherche pour le Développement/Centre National de la Recherche Scientifique, n° 5096, Centre Institut de Recherche pour le Développement, Montpellier, France; 3 Plate-forme de Proteomique Fonctionnelle, c/o Institut de Génomique Fonctionnelle, Centre National de la Recherche Scientifique Unité Mixte de Recherche 5203, Institut National de la Santé et de la Recherche Médicales, Unité 661, Université Montpellier I et II, Montpellier, France; 4 Department of Ecology and Evolutionary Biology, University of California Irvine, Irvine, California, United States of America; Albert Einstein College of Medicine, United States of America

## Abstract

We have analyzed the comportment in *in vitro* culture of 2 different genotypes of *Trypanosoma cruzi*, the agent of Chagas disease, pertaining to 2 major genetic subdivisions (near-clades) of this parasite. One of the stocks was a fast-growing one, highly virulent in mice, while the other one was slow- growing, mildly virulent in mice. The working hypothesis was that mixtures of genotypes interact, a pattern that has been observed by us in empirical experimental studies. Genotype mixtures were followed every 7 days and characterized by the DIGE technology of proteomic analysis. Proteic spots of interest were characterized by the SAMESPOT software. Patterns were compared to those of pure genotypes that were also evaluated every 7 days. One hundred and three spots exhibited changes in time by comparison with T = 0. The major part of these spots (58%) exhibited an under-expression pattern by comparison with the pure genotypes. 32% of the spots wereover-expressed; 10% of spots were not different from those of pure genotypes. Interestingly, interaction started a few minutes after the mixtures were performed. We have retained 43 different proteins that clearly exhibited either under- or over-expression. Proteins showing interaction were characterized by mass spectrometry (MALDI-TOF). Close to 50% of them were either tubulins or heat shock proteins. This study confirms that mixed genotypes of *T. cruzi* interact at the molecular level. This is of great interest because mixtures of genotypes are very frequent in Chagas natural cycles, both in insect vectors and in mammalian hosts, and may play an important role in the transmission and severity of Chagas disease. The methodology proposed here is potentially applicable to any micropathogen, including fungi, bacteria and viruses. It should be of great interest in the case of bacteria, for which the epidemiological and clinical consequences of mixed infections could be underestimated.

## Introduction

Chagas disease is a parasitic infection caused by *Trypanosoma cruzi*. Although control has improved, Chagas disease remains a serious public health problem in most Latin American countries. It is responsible for blood transfusion accidents in the United States, and it is now spreading in Europe, particularly in Spain. Available treatments are toxic and their efficiency in the chronic phase of the disease is limited. Vaccines are not available [1]. Basic research at the level of molecular mechanisms involved in parasitic infection is therefore needed to help design effective treatments and controls.

As a result of predominant clonal evolution (PCE), with occasional bouts of genetic recombination [2]–[5], *T. cruzi* natural populations are distributed into 6 major genetic subdivisions [6] or “near-clades” [4], [5]. Mixtures of genotypes pertaining to distinct near-clades are frequent in natural Chagas cycles, in the insect vector [7] as well as in the human host [8]. It has been proposed that such mixtures could play a major role in the transmission and severity of Chagas disease [9] and of other infectious diseases [9], [10].

Interactions between different *T. cruzi* genotypes have been evidenced in experimental infections of vectors [11] and laboratory mice [12]. However, these studies did not explore the molecular mechanisms involved in mixed infection interactions.

A protocol of gene expression characterization by proteomic analysis has been developed by us [13] and has made it possible to show that *T. cruzi* near-clades exhibit specific gene expression patterns.

In the present study, we have applied this protocol to the analysis of the interaction in *in vitro* cultures between two *T. cruzi* genotypes pertaining to distinct near-clades.

## Results

**Figure 1:**
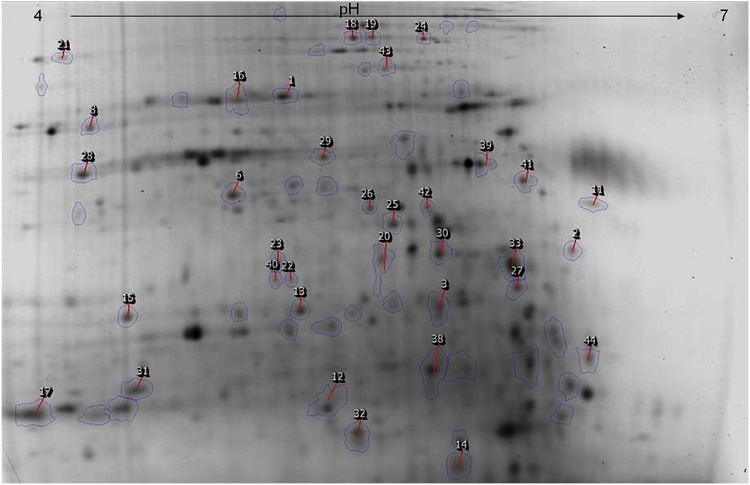
Bidimensionnal electrophoresis of the proteomic variability of the 2 *Trypanosoma cruzi* stocks P209 cl1 and Mn Cl2. The picture shows a total of 1,850 spots identified by Samespot. Circled and numbered spots correspond to the spots of interest identified by mass spectrometry. Above: PH gradient.

**Figure 2:**
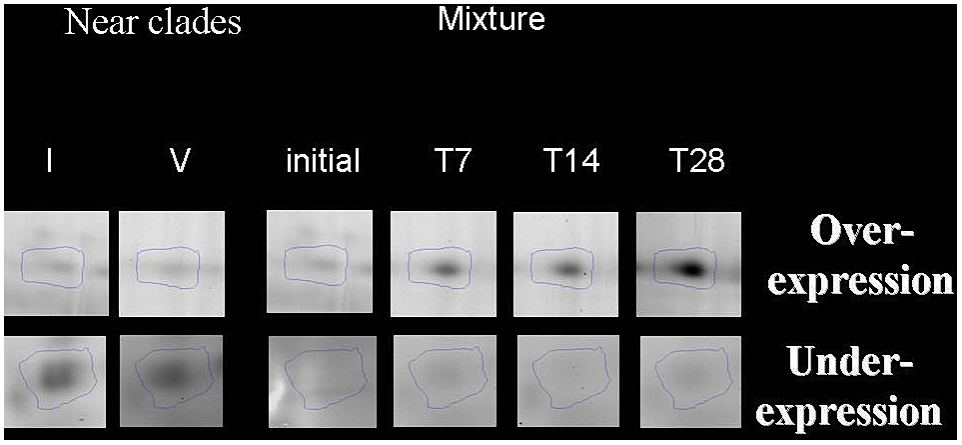
Examples of spots of interest identified by mass spectrometry. Above, over-expressed spot; below: under-expressed spot.

**Figure 3:**
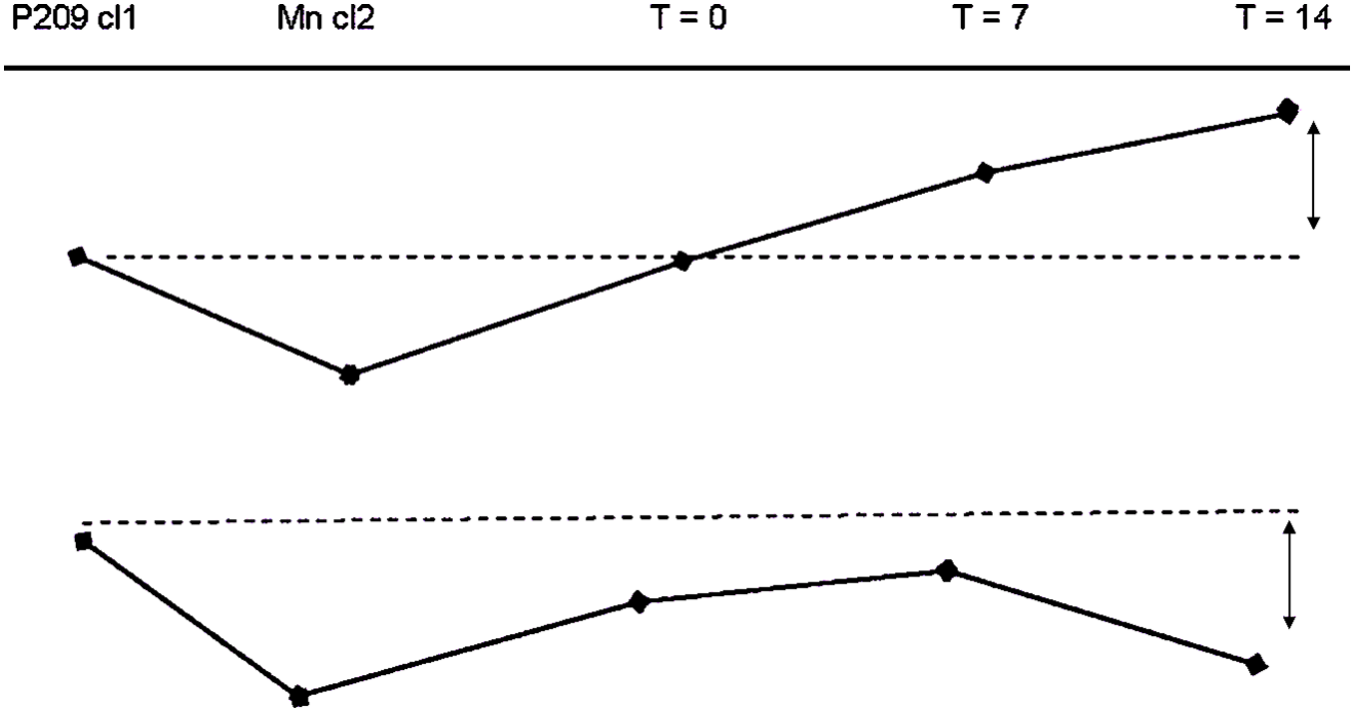
Two patterns of protein expression kinetics (from left to right): p209 cl1 (pure clone) shows the strongests spot, Mn cl2 (pure clone) shows the weakest spot; then: mixtures at different times of experiment. (a) Top row: the mixture at the end of the experiment exhibits overexpression by comparison with p209 cl1. (b) bottom row: the mixture at the end of the experiment shows underexpression by comparison with p209 cl1.

With the analysis of 2D-DIGE gels with the Progenesis SameSpots 3.1 software (Nonlinear Dynamics), we found that 103 spots exhibited change over time and were considered for further analysis. The 40 spots retained according to the 2 criteria exposed in material and methods, are shown on figure 1. Figure 2 shows 2 illustrative examples of spots of interest. A majority (58%) of the spots of interest exhibited an under-expression by comparison with the fastest genotype, while a minority (32%) showed over-expression. Figure 3 shows different patterns of protein expression kinetics, either over- or under-expression, taking as reference the dominant genotype.

The 40 spots of interest corresponded to 43 different proteins. Some spots corresponded to 2 different proteins, as evidenced by mass spectrometry.

Table 1 gives the list of protein categories identified by mass spectrometry in the spots of interest and indicates whether the proteins are either over-expressed or under-expressed.

**Table 1:** List of the proteins identified by mass spectrometry, their function and their pattern of behavior (over- vs. under-expressed) in the experiment.

Identification	Function	Identifiant	Mascot	Level expression compared to stock dominated
		Swissprot or TrEMBL	Score	
Dehydrogenase	Biosynthesis	Q2TJB7_TRYCR	165	under expression
Spermidine synthase, putative	Biosynthesis	Q4CXJ6_TRYCR	90	under expression
Tyrosine aminotransferase	Biosynthesis	ATTY_TRYCR	124	under expression
Tyrosine aminotransferase	Biosynthesis	ATTY_TRYCR	155	under expression
Urocanate hydratase, putative	Catabolism histidine	Q4D9S6_TRYCR	75	under expression
Actin	important component of thecontractile apparatus	ACT_TRYCR	91	under expression
Cysteine synthase	Metabolic process	Q4CXR9_TRYCR	83	under expression
Cysteine synthase	Metabolic process	Q4E2W1_TRYCR	129	under expression
Peroxiredoxin	Metabolic process	O79469_TRYCR (Q4CX87_TRYCR)	171	over expression
Tryparedoxin peroxidase	Metabolic process	Q8WSH1_TRYCR	141	under expression
Trypanothione reductase	Metabolic process	TYTR_TRYCR	125	under expression
Adenosylhomocysteinase	One carbon transfer metabolism	Q4D455_TRYCR	110	over expression
Adenosylhomocysteinase	One carbon transfer metabolism	Q4D455_TRYCR (Q7YUF0_TRYCR)	83	over expression
Alpha-tubulin	Polymers biosynthesis	Q26973_TRYCR	64	under expression
Elongation factor 2, putative (Fragment)	Polymers biosynthesis	Q4D5×1_TRYCR	65	under expression
Tubulin alpha chain	Polymers biosynthesis	TBA_TRYCR	80	under expression
Tubulin beta chain	Polymers biosynthesis	TBB_TRYCR	95	over expression
Tubulin beta chain	Polymers biosynthesis	TBB_TRYCR	96	over expression
Tubulin beta chain	Polymers biosynthesis	TBB_TRYCR	90	under expression
Tubulin beta chain	Polymers biosynthesis	TBB_TRYCR	184	under expression
Tubulin beta chain	Polymers biosynthesis	TBB_TRYCR	165	under expression
Tubulin beta chain	Polymers biosynthesis	TBB_TRYCR	53	under expression
Chaperonin HSP60, mitochondrial	Protein binding	CH60_TRYCR	218	under expression
Aminopeptidase, putative	Proteolysis	Q4DZJ3_TRYCR	204	over expression
Metallocarboxypeptidase	Proteolysis	Q6ZXB9_TRYCR	81	over expression
Peptidase	Proteolysis	Q4DGX6_TRYCR	74	over expression
Small GTP-binding protein Rab1, putative	Regulator	Q4CZR0_TRYCR	70	over expression
Glucose-regulated protein 78, putative	Stress response	Q4D620_TRYCR	194	under expression
Heat shock 70 kDa protein	Stress response	HSP70_TRYCR	86	over expression
Heat shock 70 kDa protein	Stress response	HSP70_TRYCR	88	under expression
Heat shock 70 kDa protein	Stress response	HSP70_TRYCR	84	under expression
Heat shock 70 kDa protein, mitochondrial	Stress response	HSP71_TRYCR	66	over expression
Heat shock 70 kDa protein, putative (Fragment)	Stress response	Q4DAZ6_TRYCR	86	over expression
Heat shock protein (HSP70)	Stress response	Q26936_TRYCR	149	under expression
Heat shock protein 85, putative (Fragment)	Stress response	Q4DKH8_TRYCR	52	over expression
Heat shock protein 85, putative (Fragment)	Stress response	Q4DKH8_TRYCR	45	under expression
Heat shock-like 85 kDa protein	Stress response	HSP85_TRYCR	104	over expression
Heat shock-like 85 kDa protein	Stress response	HSP85_TRYCR	84	under expression
Superoxide dismutase	Superoxide metabolic	Q27791_TRYCR, (Q4DI29_TRYCR)	79	over expression
I/6 autoantigen, putative	Uncharacterized	Q4DFL2_TRYCR	67	over expression
Cytochrome C oxidase subunit IV, putative	Uncharacterized	O61107_TRYCR	202	over expression
putative uncharacterized protein	Uncharacterized	QYCTC_TRYCR	75	over expression
Ribonucleoprotein p18, mitochondrial, putative	Uncharacterized	Q4CZG5_TRYCR	54	under expression

The Swissprot_TrEMBL entry is related to the reference of the protein in the Swissprot_TrEMBL database.

Score is calculated by the Mascot search engine for each identified protein matched from the MS peak list. If the Protein Score is equal to, or greater than, the Mascot Significance Level, the protein match is considered to be statistically non-random at the 95% confidence interval.

Numerous different kinds of proteins are included. The main categories are proteolysis proteins, metabolic proteins, biosynthesis proteins, structural proteins and stress proteins. However, the 2 dominant categories, which comprised about 50% of the total, were tubulins and heat shock proteins (HSPs).

It is interesting to note that some strong differences from the theoretical T = 0 (average expression of each of the 2 pure genotypes) were observed as soon as 10 mn after the actual mixing, which suggests that interaction between the 2 genotypes starts very early. This was evidenced in 76 spots out of the 103 spots that showed changing over time. Out of these 76 spots, 12 (16%) exhibited over-expression, while 64 (84%) exhibited under-expression a few minutes after mixing by comparison with the theoretical T = 0 value.

## Discussion

The goal of this study was to explore whether gene expression of *T. cruzi* genotype mixtures is different from a mere juxtaposition of the gene expression of pure genotypes. The working hypothesis was that mixed genotypes interact and exchange biochemical signals. This hypothesis is important, since mixtures of genotypes are frequent in natural Chagas cycles, both in the host [8] and in the vector [7]. Evidence of interaction in infectious disease natural cycles has been found in other pathogens. In fungi, mixtures of closely related genotypes of the same species lead to more successful transmission [14]. Mixed infections of *Plasmodium falciparum*, the agent of the most malignant form of malaria, lead to less frequent fever episodes in young subjects [15].

The present in vitro experiment dealt with 2 stocks that correspond to 2 major *T. cruzi* near-clades [4], [5]. They were selected for 3 reasons: (i) they represent « polar genotypes », phylogenetically distantly related [6]. Accordingly, their gene expression is expected to be neatly different from each other, as evidenced by proteomic analysis [13]; (ii) mixtures of these genotypes are very frequent in Chagas natural cycles [7], [8]; (iii) their behavior in *in vitro* cultures is quite different : P 209 cl1 grows much faster and is more virulent in mice than Mn cl2 [16], [17].

The present study made it possible to identify 43 different proteins that exhibit either over-expression or, more frequently, under-expression in mixed genotypes. This shows that the genotypes have interacted in these mixtures, through either reciprocal stimulation (over-expression) or inhibition (under-expression). These interactions start very early, a few minutes after the mixture was performed.

Our working hypothesis is that mixtures of genotypes play an important role in the transmission and severity of Chagas disease, through hypothetical mechanisms, which we have called « clone hitchhiking » [9]. Other authors have proposed that *T. cruzi* near-clades could have different histotropisms in the same host [18].

We do not have reliable hypotheses right now to explain why the proteins identified in the present study exhibit modified expression in genotype mixtures. The 2 categories of proteins that were most frequently identified in our study were heat shock proteins (HSPs) and tubulins. HSPs are stress proteins and their presence here could be attributed to the culture process itself. As a matter of fact, *in vitro* cultures are far from natural conditions for the parasite and obviously are a stress factor for it. This does not explain why HSPs are either over- or under-expressed in mixtures by comparison with pure genotypes, especially the fastest growing genotype P 209 cl1, which, according to previous experiments, should be overdominant in the mixtures. Tubulins are also either over- or under-expressed in mixtures. They are structural proteins of the microtubules, a major component of the cytoskeleton. Tubulins are highly involved in the motility of the flagellum [19]. It might be the case that clones interfere at the level of their motility, either to inhibit each other or to stimulate each other, for accessing nutriments.

Over- and under-expression are also observed for most proteins identified in the present study (table 1). It seems to be the case that mixture of genotypes leads to either stimulation or inhibition.

Work is in hand to explore further these phenomena through more complete collections of genotypes in *in vitro* and cell cultures, and in animal models. Further experiments with *in vitro* cultures will seek to isolate from the medium itself the hypothetical biochemical signals which mediate interactions.

We suggest that mixtures of genotypes, which have been thoroughly evidenced in Chagas natural cycles, occur also in other parasites, as well as in other pathogens [9], [10], including fungi, bacteria, and viruses. Mixtures of genotypes might play a general role in the transmission and severity of infectious diseases through clonal « hitchhiking » and cooperation [9]. The experiments reported here can potentially be applied to any pathogen to test this working hypothesis, provided that experiments are feasible. Such experiments could be especially relevant in the case of pathogenic bacteria, for which the phenomenon of mixed infection and its epidemiological and medical implications may have been underestimated.

## Materials and Methods

### *Trypanosoma Cruzi* Stocks

Two stocks were used in this study, namely P209 cl1 and Mn cl2. They belong to the *T. cruzi* genetic subdivisions or near-clades TC I, and TC V, respectively [6]. They have been cloned in the laboratory, with verification of the cloning under the microscope. Their genotype has been verified at the beginning of the study by Multilocus Enzyme Electrophoresis (MLEE) and Random Amplified Polymorphic DNA (RAPD). They have been selected for the following reasons: (i) TC I and V correspond to radically distinct groups of *T. cruzi* genotypes; (ii) mixtures of these genotypes are frequent in Chagas natural cycles [7], [8]; (iii) they exhibit very distinct phenotypes, since P209 cl1 has a faster growth *in vitro* cultures, and a higher virulence and parasitemia in mice than Mn cl2 [16], [17].

### Parasite Culture

Epimastigote stages were cultured in LIT (Liver Infusion Tryptose) with 10% fetal calf serum, in 15 ml tubes containing 1 ml of parasites for 3 ml of culture medium. Cultures were done at 27°C. Parasites were passaged every 7 days. About 2×10^6^ cells were passaged in 3 ml of fresh culture medium.

### Bulk Cultures for Proteomic Analysis

Plastic flasks of 75 cm^3^ were used for bulk cultures. After growth, 30 ml of parasite culture were transferred into 50 ml-centrifugation tubes and centrifuged at 1,500 g for 15 mn at 20°C. The supernatants were discarded and the pellets were resuspended in 10 ml of PBS (Phosphate Buffered Saline). Then the tubes were again centrifuged at 1,500 g for 15 mn at 20°C. The supernatants were discarded and the pellets were resuspended in 1 ml of PBS and transferred into 1.5 ml eppendorf tubes. The eppendorf tubes were centrifuged at 1,500 g for 10 mn at 20°C. Supernatants were discarded. Pellets were weighted and stored at −80°C until use.

### Protocol for Analysis of Mixed Stocks

Mixtures of 50% of each stock were used. Mixtures were analyzed at T = 0, T = 7 days and T = 14 days. Pure genotypes were also analyzed at T = 0, T = 7 days and T = 14 days. At T = 7 and 14, the patterns of the mixtures were compared to the patterns of P 209 cl1, which is the fastest growing strain. According to previous mixture experiments analyzed by isoenzymes and random primer amplified polymorphic DNA, P 209 cl1 becomes very fastly overdominant on MN cl2 (unpublished data). Patterns of the mixtures, if they were no interactions, should therefore be similar to the patterns of P 209 cl1. Mixtures were prepared by counting parasite cells with a Thoma chamber.

T = 0 corresponds to the moment when the mixture is done. The theoretical value taken for the mixture at T = 0 for proteomic analysis is the average value of the 2 genotypes considered separately. However, as we will expose further, interaction between the genotypes starts as early as a few minutes after the mixture is made. We, therefore, give the proteomic results of the mixture a few minutes after T = 0.

Three replicates were performed for each measure.

### Proteomic Analysis

Bidimensional electrophoresis DIGE (Difference Gel Electrophoresis; 2D-DIGE) was used. This technique relies on the use of 3 fluorochromes or CyDyes (Cy2, Cy3 and Cy5), which bind themselves to lysine aminoacids.

#### Protein extraction

Parasite pellets were resuspended into 500 µl of a solubilization buffer solution, then the parasite cell walls were lysed by immersion in liquid nitrogen 3 times for a few seconds each time. Benzonase Nuclease was then added in order to degrade DNA and RNA in the samples. Samples were then centrifuged at 20,000 g for 15 mn at 4°C. PH was then adjusted at 8.5.

#### Protein labeling

Labeling with fluorochromes was performed with the CyDye DIGE fluors kit (minimal dyes) of ETTAN DIGE (Amersham Biosciences).

According to the manufacturer’s recommendations, 50 µg of proteins were labeled with 400 pmol of marker. After labeling, samples were kept in the dark for 30 mn. Reaction was then stopped by saturating the solution with 1 µl of lysine. Samples were then put again in the dark for 15 mn.

#### Protein separation by 2-dimensional electrophoresis (2D-PAGE)

(i)1st dimension: separation by isoelectrofocalization (IEF). IEF separates proteins according to their isoelectric point (IP). IEF separation was performed on strips (IPG-strips, Amersham) with a PH gradient of 4 to 7. Then, the strips were rehydrated with 450 µl of rehydration buffer covered with mineral oil to avoid dehydration (Drystrip Cover Fluid, Amersham). Proteins were then isoelectrofocused at 30V for 3 h, then at 30V to 1000 V for 5 h, then from 1000V to 8,000 V for 4 h, and finally to 8,000 V for 5 h before coming back to 30 V again. (ii) 2^nd^ dimension: SDS-PAGE (Sodium Dodecyl Sulfate Polyacrylamide Gel Electrophoresis). SDS-PAGE separates proteins according to their molecular weight (MW). After IEF, the proteins contained in the strips were equilibrated in a buffer containing SDS (Sodium Dodecyl Sulfate), allowing the proteins to be negatively charged, so that they have the same electric charge and MW is the only parameter acting in SDS-PAGE. Gels were then set in a tank (Ettan Dalt II system, Amersham). Electrophoresis was performed at 20 mA/gel overnight at 4°C.

Gels were then revealed by using a scanner (Typhoon Variable Mode Imager 9400). For each gel, pictures were checked by the ImageQuant software (GE Healthcare Life Sciences).

#### Selection of spots of interest

It was performed by comparing proteomic profiles with the Samespot software (Nonlinear Dynamics), which is specifically designed to assess the reproducibility of changes in protein levels determined by 2-D DIGE. The first step aimed to compare all spots at times T = 0, 7 and 14 days. The null hypothesis was that there is no significant protein expression change over time. Spots that differed significantly from that of the theoretical T = 0 at p≤0.05 were selected. The second step aimed to compare these spots to the spots of each of the pure genotypes. The null hypothesis was here that protein expression of the mixture should be similar to that of the genotype that has the fastest growth rate, i.e. P209 cl1 (see above). The spots of interest that were finally retained followed these 2 criteria: (i) change over time; (ii) protein expression different from the fastest growing genotype. For material reasons, we finally retained only 40 spots, which proved to correspond to 43 proteins.

#### Identification by mass spectrometry

After coloration of the spots by Coomiassie G-250 blue, out of 1,850 spots identified by Samespot, the 40 spots of interest were removed from the gels. Enzymatic in-gel digestion was performed automatically (Tecan freedom evo proteomics) according to Shevchenko et al’s modified protocol [20]. Briefly, protein spots were digested using 150 ng of trypsin, peptide extraction was performed using 5 sonication cycles of 2 mn each, and peptides were concentrated 1 hr at 50°C in a heat block. Peptide samples were automatically spotted (Tecan freedom evoH proteomics). For this step, 0.5 ml of peptide sample and 0.5 ml of alpha-cyano-4-hydroxy-trans-cinnamic acid (a saturated solution prepared in acetonitrile/trifluoroacetic acid, 50∶0.1%, vortexed, sonicated 30 s and microcentrifuged 30 s with a 1/3 dilution of the supernatant used as the matrix) were deposited on a 384-well MALDI anchorship target using the drydroplet procedure [21] and air dried at room temperature. Peptide samples were then desalted using a 10 mM phosphate buffer and dried again at room temperature. Peptides samples were then analyzed by mass spectrometry MALDI-TOF.

MALDI-TOF MS analysis was performed using UltraFlex MALDI TOF-TOF mass spectrometer (Brucker Daltonics, Bremen, Germany) in the reflectron mode with a 26 kV accelerating voltage and a 50 ns delayed extraction. The AutoXecute module of Flexcontrol v3.0 (Bruker Daltonics) (laser power ranged from 32 to 40%, 600 shots) was used to acquire mass spectra. Spectra were analyzed using FlexAnalysis software v3.0 (Bruker Daltonics) and calibrated internally with the autoproteolysis peptides of trypsin (m/z: 842.51; 1045.556; 2211.10). Peptides were selected in the mass range of 900–3000 Da.

Peptide Mass Fingerprint identification of proteins was performed by searching against the *Trypanosoma* entries of either the Swiss-Prot or TrEMBL databases (v20090506, http://www.uniprot.org/) and by using the MASCOT v2.2.05 algorithm (Matrix Science Inc. http://www.matrixscience.com/) with trypsin enzyme specificity and one trypsin missed cleavage allowed. Carbamidomethyl was set as fixed cystein modification and oxidation was set as variable methionine modification for searches. A mass tolerance of 50 ppm was allowed for identification. Matching peptides with one missed cleavage were considered as pertinent when there were two consecutive basic residues or when arginine and lysine residues were in an acidic context. MASCOT scores higher than 57 were considered as significant (p<0.05) for Swiss-Prot and TrEMBL database interrogations.
